# Cathepsin B Regulates Collagen Expression by Fibroblasts via Prolonging TLR2/NF-*κ*B Activation

**DOI:** 10.1155/2016/7894247

**Published:** 2016-08-28

**Authors:** Xue Li, Zhou Wu, Junjun Ni, Yicong Liu, Jie Meng, Weixian Yu, Hiroshi Nakanishi, Yanmin Zhou

**Affiliations:** ^1^Department of Implantology, School of Stomatology, Jilin University, Changchun 130021, China; ^2^Department of Aging Science and Pharmacology, Faculty of Dental Science, Kyushu University, Fukuoka 812-8582, Japan; ^3^OBT Research Center, Faculty of Dental Science, Kyushu University, Fukuoka 812-8582, Japan

## Abstract

Fibroblasts are essential for tissue repair due to producing collagens, and lysosomal proteinase cathepsin B (CatB) is involved in promoting chronic inflammation. We herein report that CatB regulates the expression of collagens III and IV by fibroblasts in response to a TLR2 agonist, lipopolysaccharide from* Porphyromonas gingivalis* (*P.g.* LPS). In cultured human BJ fibroblasts, mRNA expression of CatB was significantly increased, while that of collagens III and IV was significantly decreased at 24 h after challenge with* P.g.* LPS (1 *μ*g/mL). The* P.g.* LPS-decreased collagen expression was completely inhibited by CA-074Me, the specific inhibitor of CatB. Surprisingly, expression of collagens III and IV was significantly increased in the primary fibroblasts from CatB-deficient mice after challenge with* P.g.* LPS. The increase of CatB was accompanied with an increase of 8-hydroxy-2′-deoxyguanosine (8-OHdG) and a decrease of I*κ*B*α*. Furthermore, the* P.g.* LPS-increased 8-OHdG and decreased I*κ*B*α* were restored by CA-074Me. Moreover, 87% of CatB and 86% of 8-OHdG were colocalized with gingival fibroblasts of chronic periodontitis patients. The findings indicate the critical role of CatB in regulating the expression of collagens III and IV by fibroblasts via prolonging TLR2/NF-*κ*B activation and oxidative stress. CatB-specific inhibitors may therefore improve chronic inflammation-delayed tissue repair.

## 1. Introduction

Fibroblasts are essential for tissue repair due to their activity in producing collagens. Collagen can be divided into fibrillar and nonfibrillar families. Collagens I and III, the major fibrillar collagens in connective tissues, play important roles in tissue repair [1]. In particular, collagen III is suggested to regulate collagen I synthesis [2], as a higher ratio of collagen III to collagen I is associated with scarless wound healing in mammal models [3, 4], and collagen III-deficient mice exhibit more pronounced wound contracture than those with collagen III [5]. However, the expression of collagen III is regulated by the activity of local fibroblasts [6]. Collagen IV, a nonfibrillar collagen, is a major component of the basement membrane (BM) [7, 8]. Recently, collagen IV has been shown to participate in the innate immune response by regulating cellular adhesion and migration [9], and Col4a1 (the human collagen IV gene) mutation-associated phenotypic features include chronic inflammation and immune activation [10–12].

Toll-like receptors (TLRs), which are expressed in fibroblasts [13, 14], have been observed to modulate tissue repair during chronic inflammation [15–17]. Furthermore, nuclear factor kappa B (NF-*κ*B), the key transcription factor of TLR signaling, has been shown to inhibit collagen I gene expression directly [18] and mediate oxidative stress-decreased collagen biosynthesis [19].

Cathepsin B (CatB; EC 3.4.22.1), a typical cysteine lysosomal protease, promotes inflammation involved in the production of mature IL-1*β* [20–23]. CatB was recently found to be responsible for NF-*κ*B activation through autophagy degradation of inhibitor of *κ*B*α* (I*κ*B*α*) in microglia/macrophages [24]. Naturally, as a protease, CatB degrades collagens in fibroblasts [25], but the role of CatB in regulating collagen expression by fibroblasts during chronic inflammation is still unknown.

In the present study, we aimed to elucidate the involvement of CatB in the expression of collagens III and IV by fibroblasts after challenge with a TLR2 agonist, lipopolysaccharide from* Porphyromonas gingivalis* (*P.g.* LPS).

## 2. Materials and Methods

### 2.1. Reagents

*P.g. *LPS was purchased from InvivoGen (San Diego, CA, USA). Bay 11-7082 and SN50, the specific NF-*κ*B inhibitors, were purchased from Sigma-Aldrich (St. Louis, MO, USA). CA-074Me, the specific CatB inhibitor, was purchased from Peptide Institute. Inc. (Osaka, Japan). Mouse anti-8-OHdG (N213120) was purchased from NOF Corporation (Kyoto, Japan). Antibodies of rabbit anti-fibronectin (H-300), goat anti-CatB (S-12), and rabbit anti-I*κ*B*α* were purchased from Santa Cruz Biotechnology (Santa Cruz, CA, USA). Mouse anti-TLR2 (T 2.5) was purchased from eBioscience (San Diego, CA, USA). Mouse anti-Collagen III (Col-29) and mouse anti-Collagen IV (COL-94) were purchased from Abcam (Cambridge, UK).

### 2.2. Tissue Preparation from Periodontitis Patients

The gingival tissues were obtained from patients with periodontal surgery or extraction. The periodontal diagnosis of subjects with chronic periodontitis was established based on the clinical and radiographic criteria defined at the 1999 International World Workshop for a Classification of Periodontal Diseases and Conditions [26]. The samples included 7 cases diagnosed as chronic periodontitis (36–60 years of age, 3 males and 4 females), which were obtained from the Periodontology Department of the School of Stomatology, Jilin University. After periodontal surgery, the excised gingival specimens were immediately placed in liquid nitrogen and subsequently frozen at −80°C until use in experiments.

The gingival samples were immersed in a periodate lysine paraformaldehyde (PLP) fixative consisting of 0.01 M sodium metaperiodate, 0.075 M L-lysine-HCL, 4% paraformaldehyde, and 0.03% phosphate buffer (pH 6.2) for 6 h at 4°C. The specimens were protected for 2 days in 30% sucrose in phosphate-buffered saline (PBS) and then embedded in an optimal cutting temperature compound (Sakura Finetechnical Co., Ltd., Tokyo, Japan). The coronal frozen sections (thickness of 8 *μ*m) were subjected to the immunohistochemical analyses.

### 2.3. BJ Human Fibroblast Cell Line Culture

BJ (CRL-2522) cells purchased from ATCC (Manassas, VA, USA) were cultured in Minimum Essential Medium (MEM; GIBCO, Grand Island, NY, USA) supplemented with 10% fetal bovine serum (FBS, GIBCO), 1% penicillin-streptomycin, 10 *μ*g/mL insulin, and 450 mg/mL glucose. The cells were cultured at 37°C in a humidified atmosphere with 5% CO_2_.

### 2.4. Primary CatB-Deficient (CatB^−/−^) Fibroblast Culture

Three-day-old CatB^−/−^ mice were sacrificed using an excessive amount of ether. The skin tissue was removed and immediately washed first in alcohol (75%) and then in PBS. The tissue was then cut and transferred into a 6-well plate. The fibroblasts were grown in MEM supplemented with 10% FBS, 1% penicillin-streptomycin, 10 *μ*g/mL insulin, and 450 mg/mL glucose in a humidified atmosphere with 5% CO_2_ at 37°C. The medium was changed after five days, and then the cells were cultivated with culture medium in a new plate. The experiments were performed using early passage fibroblasts before the second passage.

### 2.5. Determination of Cell Viability

BJ cells were cultured in a 96-well plate for 24 h (5 × 10^3^ cells/well) and then incubated with various concentrations of* P.g.* LPS for 48 h. Cell viability was assessed using the cell-counting kit-8 (CCK-8; Dojindo, Kumamoto, Japan) in accordance with the manufacturer's instructions, as follows: After the treatment of* P.g.* LPS, 10 *μ*L CCK-8 was added to each well of the 96-well plate and then incubated at 37°C for 2 h. In accordance with the instructions, the optical density was read at a wavelength of 450 nm using the microplate reader. Cell viability was calculated using the following formula: optical density of treated group/control group × 100%.

### 2.6. Immunofluorescence Imaging

The coronal frozen sections of periodontitis tissues were incubated with antibody diluent overnight at 4°C. The sections were then treated with primary rabbit anti-fibronectin (H-300, 1 : 1000), mouse anti-8-OHdG (N213120, 1 : 1000), goat anti-CatB (S-12, 1 : 1000), and mouse anti-TLR2 (T 2.5, 1 : 1000) for 12 h at 4°C. After washing with PBS, the sections were incubated with a mixture of FITC-conjugated and rhodamine-conjugated secondary antibodies for 2 h at 24°C. The sections were then washed again with PBS and mounted in Vectashield antifading medium (Vector Laboratory, CA, USA) and examined using a confocal laser scanning microscope (CLSM, C2si, Nikon, Japan). The CLSM images of individual sections were taken as a stack at a 1-*μ*m step size in the *z*-direction with 20x objectives (numerical aperture = 0.5), zoom factor 1.0. A rectangle (1024 × 1024 pixels) corresponding to the size of 450 × 450 *μ*m was used as the counting frame. The CLSM images were shown as the middle of the stacked images.

For the cultured BJ fibroblasts staining, the cells were seeded at 5 × 10^5^ cells/mL in 24-well plates. 48 h after* P.g.* LPS (1 *μ*g/mL) challenge or pretreatment with CA-074Me, the cells were fixed with 4% paraformaldehyde and then incubated with the mouse anti-p65 (1 : 500) and mouse anti-8-OHdG (1 : 1000) overnight at 4°C. After being incubated with anti-mouse Alexa 488 (1 : 1000, Jackson Immunoresearch Lab. Inc.) at room temperature for 2 h, they were then incubated with Hoechst (1 : 200, Sigma-Aldrich, Japan) and mounted in the antifading medium Vectashield. Fluorescence images were taken using a CLSM (C2si, Nikon, Japan).

### 2.7. Real-Time Quantitative Polymerase Chain Reaction Analysis (RT-qPCR)

BJ cells were treated with* P.g.* LPS (1 *μ*g/mL) for 3, 12, 24, and 48 h; primary CatB^−/−^ fibroblast cells and primary wild type fibroblasts were treated with* P.g.* LPS (1 *μ*g/mL) for 24 and 48 h. After treatment, the cells were collected. A set of the BJ cells was pretreated with Bay 11-7082 (20 *μ*M, [27]), CA-074Me (50 *μ*M, [22]), or SN50 (20 *μ*M) for 1 h and then treated with* P.g.* LPS (1 *μ*g/mL), incubated for 48 h, and harvested.

mRNA isolated from these cells was subjected to RT-qPCR. The total RNA was extracted with the Purelink RNA microkit (Invitrogen, Tokyo, Japan) in accordance with the manufacturer's instructions. A total of 1000 ng of extracted RNA was reverse transcribed to cDNA using the High Capacity RNA-to-cDNA Master Mix (Applied Biosystems, Foster City, CA, USA). The thermal cycling was held at 95°C for 5 min, followed by 40 cycles at 95°C for 5 sec and at 60°C for 10 sec. The cDNA was amplified in duplicate using TaqMan Universal PCR Master Mix (Applied Biosystems) with an Applied Biosystems 7500/7500 Fast Real-Time PCR System. The data were evaluated using the 7500 software program (version 2.0, Applied Biosystems). The primer sequences used were as follows: Human Actin: 5′-AGA GCT ACG AGC TGC CTG AC-3′ and 5′-AGC ACT GTG TTG GCG TAC AG-3′; Human TLR2: 5′-GCC AAA GTC TTG ATT GAT TGG-3′ and 5′-TTG AAG TTC TCC AGC TCC TG-3′; Human Collagen III: 5′-TGG TGT TGG AGC CGC TGC CA-3′ and 5′-CTC AGC ACT AGA ATC TGT CC-3′; Human Collagen IV: 5′-ATG TCA ATG GCA CCC ATC AC-3′ and 5′-CTT CAA GGT GGA CGG CGT AG-3′; Human CatB: 5′-TGA CGT GTT GGT ACA CTC CTG-3′ and 5′-TGG AGG GAG CTT TCT CTG TG-3′; Mouse Actin: 5′-CAA TAG TGA TGA CCT GGC CGT-3′ and 5′-AGA GGG AAA TCG TGC GTG AC-3′; Mouse Collagen III: 5′-CCA GCT GGG CCT TTG ATA CCT-3′ and 5′-TGC CCA CAG CCT TCT ACA CCT-3′; and Mouse Collagen IV: 5′-AGG CAG GTC AAG TTC TAG CG-3′ and 5′-CAA GCA TAG TGG TCC GAG TC-3′. For data normalization, an endogenous control (actin) was assessed to control for the cDNA input, and the relative units were calculated by a comparative Ct method. All of the RT-qPCR experiments were repeated three times, and the results are presented as the means of the ratios ± the standard error of the mean (SEM).

### 2.8. Electrophoresis and Immunoblotting

BJ cells were cultured in the 6-well plate at a density of 5 × 10^5^ cells/mL. After treatment with* P.g.* LPS (1 *μ*g/mL) for 12, 24, and 48 h, the cells were collected for experiments. A set of the BJ cells was pretreated with CA-074Me (50 *μ*M) for 1 h, and then* P.g.* LPS (1 *μ*g/mL) was added to the medium. The cells continued to be cultured for 48 h and were then harvested.

The cells were electrophoresed in 7.5% or 12% SDS-polyacrylamide gels, and the proteins on the SDS gels were transferred electrophoretically to nitrocellulose membranes, which were washed with PBS and then blocked for 1 h. The membranes were incubated with one of the following primary antibodies overnight at 4°C: rabbit anti-I*κ*B*α* (1 : 1000), mouse anti-TLR2 (1 : 1000), rabbit anti-collagen III (1 : 500), rabbit anti-collagen IV (1 : 500) or goat anti-CatB (1 : 1000). After washing the membranes with PBS, the membranes were incubated with horseradish peroxidase- (HRP-) labeled anti-rabbit (1 : 1000, GE Healthcare, UK), anti-mouse (1 : 1000, GE Healthcare, UK), or anti-goat (1 : 1000, GE Healthcare, UK) antibodies for 2 h at 24°C, and then the protein bands were detected by an enhanced chemiluminescence detection system (ECK kit, Thermo Scientific, Rockford, IL, USA) using an image analyzer (LAS-4000; Fuji Photo Film, Tokyo, Japan).

### 2.9. 8-OHdG Assay

BJ cells were cultured in the 10 cm dish at a density of 5 × 10^5^ cells/mL. After the cells adhering to the bottom of the dish,* P.g.* LPS (1 *μ*g/mL) was treated or pretreated with CA-074Me (50 *μ*M) for 1 h. After 48 h, the cells were collected and subjected to DNA extraction by DNA Extractor TIS Kit (Wako, Osaka, Japan) according to the manufacturer's protocol. The extracted DNA were calculated and prepared according to 8-OHdG Assay Preparation Reagent Set (Wako, Osaka, Japan), 200 *μ*g/150 *μ*L of each sample were heated at 98°C for 2 min. After chill in ice for 5 min, the samples were incubated with 19 *μ*L acetic buffer and 10 *μ*L of nuclease P1 solution at 37°C for 30 min. 20 *μ*L of Tris buffer and 1 *μ*L of Alkaline Phosphatase solution were added and incubated at 37°C for 30 min. Then, the samples were subjected to 8-OHdG ELISA kit (High sensitive-8-OHdG check; Japan Institute for the Control of Aging, Fukuroi, Japan) following the manufacturer's protocol.

### 2.10. Data Analysis

The data are represented as the means ± SEM. The statistical analyses were performed using a one- or two-way analysis of variance (ANOVA) with a post hoc Tukey's test using the GraphPad Prism software package (GraphPad Software Inc., San Diego, CA, USA). A value of *P* < 0.05 was considered to indicate statistical significance.

## 3. Results

### 3.1. The Expression of CatB as well as Collagen III and Collagen IV after* P.g.* LPS Challenge

**Figure 1:**
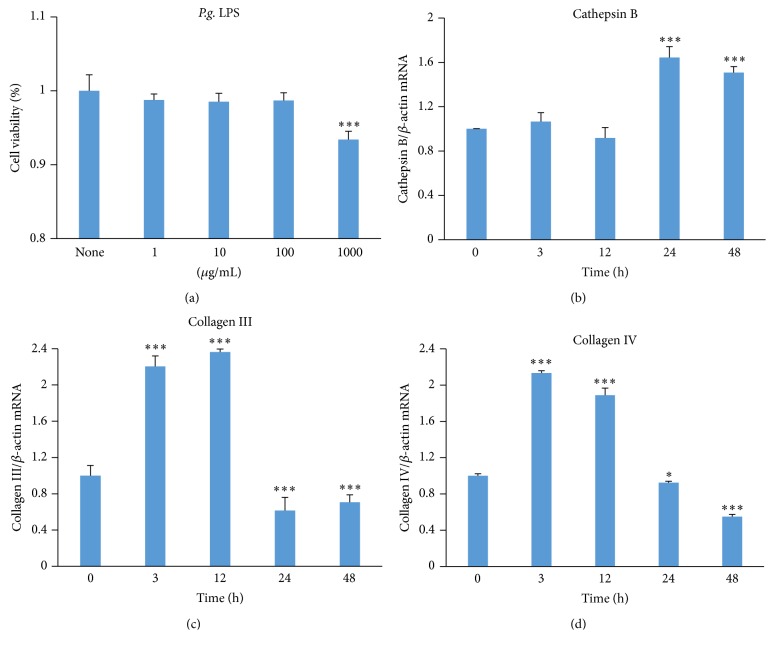
Expression of CatB and collagens III and IV by BJ fibroblasts after challenge with* P.g.* LPS. (a) The cell viability of BJ fibroblasts at 48 h after challenge with different dose of* P.g.* LPS by using cell-counting kit-8. (b) The mean mRNA expression level of CatB (3, 12, 24, and 48 h) after challenge with* P.g.* LPS (1 *μ*g/mL). (c) The mean mRNA expression level of collagen III (3, 12, 24, and 48 h) after challenge with* P.g.* LPS (1 *μ*g/mL). (d) The mean mRNA expression level of collagen IV (3, 12, 24, and 48 h) after challenge with* P.g.* LPS (1 *μ*g/mL). Each column and bar represent the mean ± SEM (*n* = 4 each). The asterisks indicate a statistically significant difference from the value at the start of experiments (0 h) (^*∗*^ *P* < 0.05, ^*∗∗∗*^ *P* < 0.001).

First, we examined the suitable concentrations of* P.g.* LPS for challenging the cultured human BJ fibroblasts. The cell viability of BJ fibroblasts decreased significantly at 48 h after challenge with 1000 *μ*g/mL* P.g.* LPS (Figure 1(a)). We therefore decided to use the concentration of* P.g.* LPS at 1 *μ*g/mL in subsequent experiments, which was one-thousandth of the concentration that reduced the cell viability for the time course of the experiments.

During the time courses (3, 12, 24, and 48 h) challenged with* P.g.* LPS (1 *μ*g/mL), the mean mRNA expression of CatB was significantly increased at 24 and 48 h (the late culture periods) but not at 3 or 12 h (the early culture periods) compared with the unchallenged cells (Figure 1(b)). In contrast, the mean mRNA expression of collagens III and IV was significantly increased at 3 and 12 h but was significantly decreased at 24 and 48 h after challenge with* P.g.* LPS compared with the unchallenged cells (Figures 1(c) and 1(d)). These observations provided the first evidence for a negative link between CatB and collagens III and IV during chronic activation of TLR2 signaling.

### 3.2. The Regulation of Collagens III and IV Expression by CatB in Fibroblasts after* P.g.* LPS Challenge

We next examined the roles of CatB in regulating the expression of collagens III and IV after challenge with* P.g.* LPS via two approaches: pharmacological inhibition using the specific CatB inhibitor CA-074Me and genetic deletion of CatB using primary fibroblasts from CatB-deficient (CatB^−/−^) mice.

**Figure 2:**
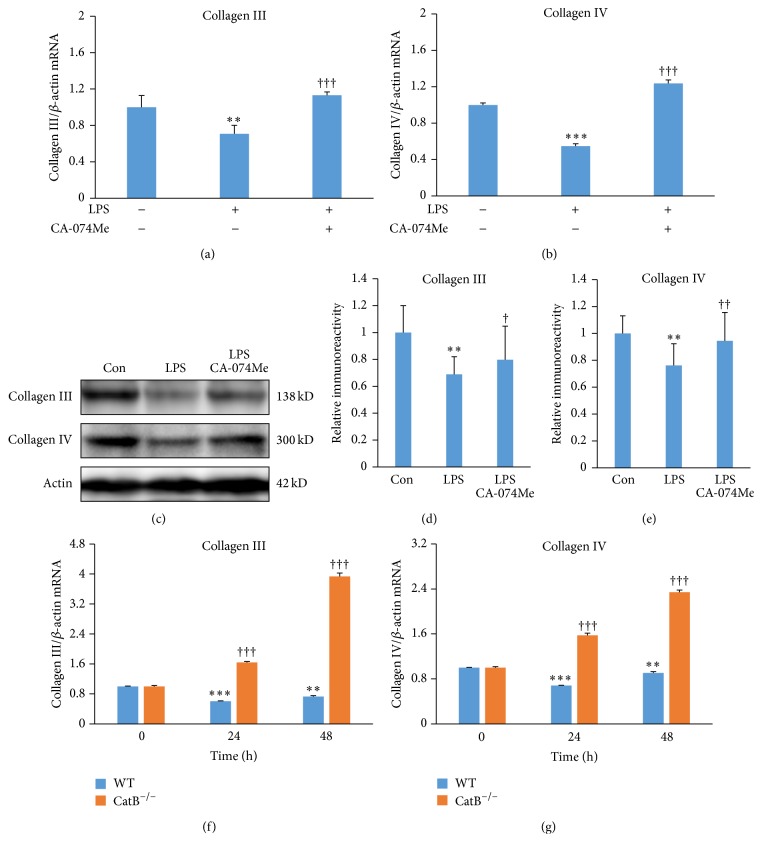
The effects of CatB on the expression of collagens III and IV by BJ fibroblasts after challenge with* P.g.* LPS. (a) The effect of CA-074Me (50 *μ*M, 1 h before* P.g. *LPS challenge) on the expression of collagen III at 48 h after challenge with* P.g. *LPS (1 *μ*g/mL). (b) The effect of CA-074Me on the expression of collagen IV at 48 h after challenge with* P.g.* LPS (1 *μ*g/mL). Each column and bar represent the mean ± SEM (*n* = 4 each). The asterisks indicate a statistically significant difference from the value in untreated cells (^*∗∗*^ *P* < 0.01, ^*∗∗∗*^ *P* < 0.001). The crosses indicate a statistically significant difference from the value in* P.g.* LPS-challenged cells without pretreatment with CA-074Me (^†††^ *P* < 0.001). (c) The effect of CA-074Me on the protein expression of collagen III and collagen IV at 48 h after challenge with* P.g.* LPS (1 *μ*g/mL). ((d), (e)) The quantitative analyses of the immunoblotting for collagen III (d) and collagen IV (e). Each column and bar represent the mean ± SEM (*n* = 4 each). The asterisks indicate a statistically significant difference from the value in untreated cells (^*∗∗*^ *P* < 0.01). The crosses indicate a statistically significant difference from the value in* P.g.* LPS-challenged cells without pretreatment with CA-074Me (^†^ *P* < 0.05, ^††^ *P* < 0.01). (f) The expression of collagen III in the primary fibroblasts from wild type and CatB^−/−^ mice at 24 and 48 h after challenge with* P.g.* LPS (1 *μ*g/mL). (g) The expression of collagen IV in the primary fibroblasts from wild type and CatB^−/−^ mice at 24 and 48 h after challenge with* P.g.* LPS (1 *μ*g/mL). Each column and bar represent the mean ± SEM (*n* = 4 each). The asterisks indicate a statistically significant difference from the value in unchallenged cells from wild type mice (^*∗∗*^ *P* < 0.01, ^*∗∗∗*^ *P* < 0.001). The crosses indicate a statistically significant difference from the value in the unchallenged cells from CatB^−/−^ mice (^†††^ *P* < 0.001).

Pretreatment with CA-074Me (50 *μ*M, 1 h) was able to restore the mean mRNA expression of both collagens III and IV to control levels at 48 h after challenge with* P.g.* LPS (Figures 2(a) and 2(b)). The protein expression levels of collagens III and IV were also restored by pretreatment with CA-074Me (50 *μ*M, 1 h) after 48 h challenge with* P.g.* LPS (Figures 2(c)–2(e)). In the primary fibroblasts from wild type mice, the mean mRNA expression of collagens III and IV was significantly decreased at 24 and 48 h after challenge with* P.g.* LPS, findings which were consistent with those observed in human BJ fibroblasts (Figures 1(c) and 1(d)). To our surprise, the mean mRNA expression of collagens III and IV in the primary fibroblasts from CatB^−/−^ mice was significantly increased compared with that of wild type mice at 24 and 48 h after challenge with* P.g. *LPS (Figures 2(f) and 2(g)). These observations clearly demonstrate that CatB has a critical role in regulating the expression of collagens III and IV by fibroblasts during chronic activation of TLR2 signaling.

### 3.3. The Regulation of NF-*κ*B Activation and Oxidative Damage for Decreasing Collagens III and IV by CatB after* P.g.* LPS Challenge

**Figure 3:**
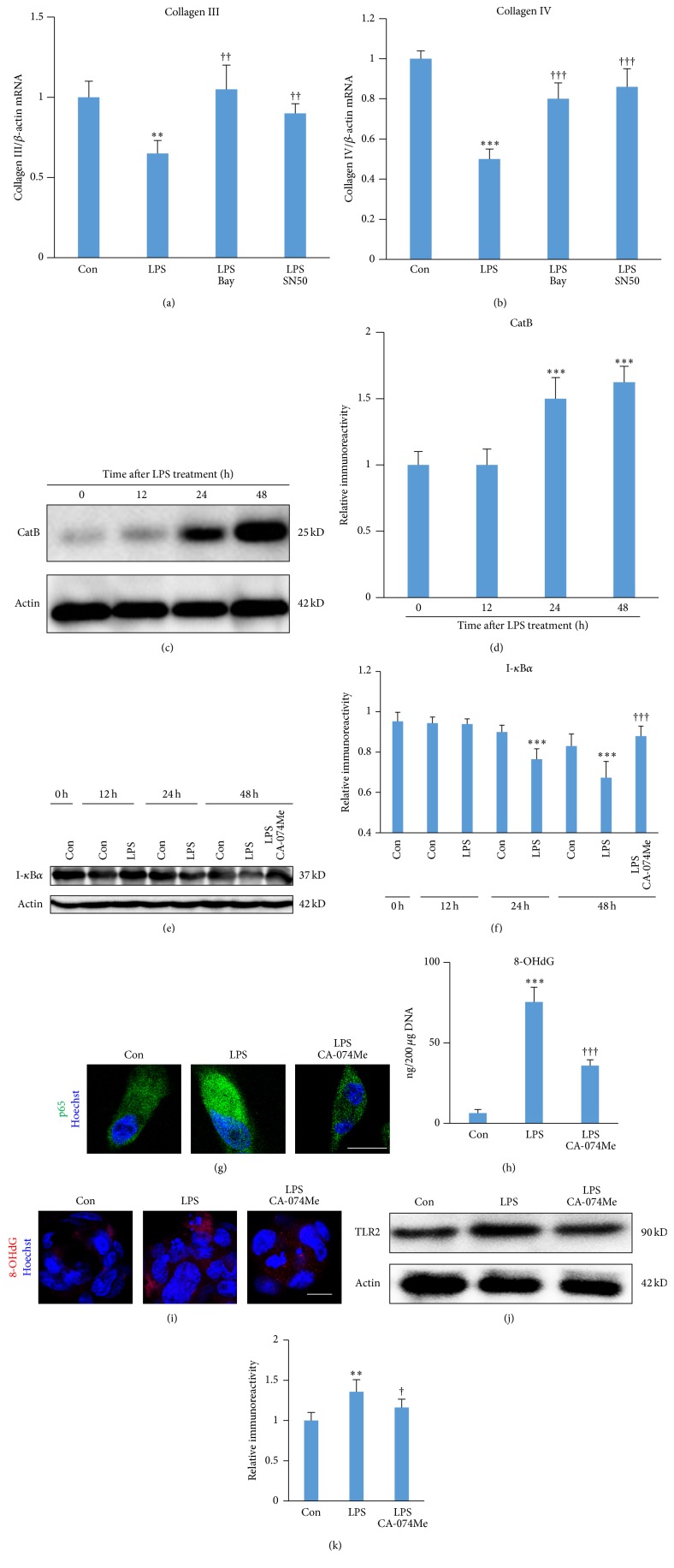
The effects of CatB on NF-*κ*B activation and oxidative damage in BJ fibroblasts after challenge with* P.g.* LPS. ((a), (b)) The effect of Bay 11-7082 (Bay, 20 *μ*M, 1 h before* P.g.* LPS challenge) and SN50 (20 *μ*M, 1 h before* P.g.* LPS challenge) on the expression of collagen III (a) and collagen IV (b) at 48 h after challenge with* P.g. *LPS (1 *μ*g/mL). Each column and bar represent the mean ± SEM (*n* = 4 each). The asterisks indicate a statistically significant difference from the value in unchallenged cells (^*∗∗*^ *P* < 0.01, ^*∗∗∗*^ *P* < 0.001). The crosses indicate a statistically significant difference from the value in* P.g.* LPS-challenged cells without pretreatment with Bay 11-7082 (^††^ *P* < 0.01, ^†††^ *P* < 0.001). (c) The time course of the protein expression of CatB in BJ fibroblasts after challenge with* P.g.* LPS (1 *μ*g/mL). (d) The quantitative analyses of the immunoblotting for CatB. Each column and bar represent the mean ± SEM (*n* = 4, each). The asterisks indicate a statistically significant difference from the value in the unchallenged cells (^*∗∗∗*^ *P* < 0.001). (e) The time course of the protein expression of I*κ*B*α* in BJ fibroblasts after challenge with* P.g.* LPS (1 *μ*g/mL). (f) The quantitative analyses of the immunoblotting for I*κ*B*α*. Each column and bar represent the mean ± SEM (*n* = 4, each). The asterisks indicate a statistically significant difference from the value in the unchallenged cells (^*∗∗∗*^ *P* < 0.001). The crosses indicate a statistically significant difference from the value in 48 h* P.g.* LPS-challenged cells without pretreatment with CA-074Me (^†††^ *P* < 0.001). (g) The immunofluorescent CLSM images of p65 (green) in BJ fibroblasts after 48 h challenged with* P.g.* LPS or pretreatment with CA-074Me. Scale bar = 10 *μ*m. (h) The amount of 8-OHdG in BJ fibroblasts 48 h after challenge with* P.g.* LPS. Each column and bar represent the mean ± SEM (*n* = 4, each). The asterisks indicate a statistically significant difference from the value in the unchallenged cells (^*∗∗∗*^ *P* < 0.001). The crosses indicate a statistically significant difference from the value in 48 h* P.g.* LPS-challenged cells without pretreatment with CA-074Me (^†††^ *P* < 0.001). (i) The immunofluorescent CLSM images of 8-OHdG (green) in BJ fibroblasts after 48 h challenged with* P.g.* LPS or pretreatment with CA-074Me. Scale bar = 10 *μ*m. (j) The protein expression of TLR2 in BJ fibroblasts 48 h after challenge with* P.g.* LPS (1 *μ*g/mL). (k) The quantitative analyses of the immunoblotting for TLR2 in (j). Each column and bar represent the mean ± SEM (*n* = 4, each). The asterisks indicate a statistically significant difference from the value in the unchallenged cells (^*∗∗*^ *P* < 0.01). The crosses indicate a statistically significant difference from the value in 48 h* P.g.* LPS-challenged cells without pretreatment with CA-074Me (^†^ *P* < 0.05).

Next, we investigated the molecular mechanisms by which CatB regulates the expression of collagens III and IV by fibroblasts after* P.g.* LPS challenge. Pretreatment with Bay 11-7082 or SN50, the specific inhibitors of NF-*κ*B (1 h before* P.g.* LPS challenge), was able to inhibit the* P.g.* LPS-induced decrease in the mean mRNA expression of collagens III and IV by BJ fibroblasts at 48 h (Figures 3(a) and 3(b)), demonstrating that the* P.g. *LPS-induced decrease in the collagens was dependent on NF-*κ*B activation. These results were consistent with those of a previous report showing that collagen I gene expression was dependent on NF-*κ*B activation [18].

We then further analyzed the expression of CatB, I*κ*B*α*, and 8-OHdG (a critical biomarker of oxidative stress). The mean expression of CatB was significantly increased at 24 and 48 h (Figures 3(c) and 3(d)), while the mean expression of I*κ*B*α* was significantly decreased compared with that of the control group at 24 and 48 h after* P.g.* LPS challenge (Figures 3(e) and 3(f)). The mean expression of the* P.g.* LPS-decreased I*κ*B*α* was paralleled with the significant increase in the nuclear localization of p65 NF-*κ*B at 24 after* P.g. *LPS challenge (Figure 3(g)). The mean amount of 8-OHdG was significantly increased at 48 h (Figure 3(h)) and the protein expression level of TLR2 was also increased at 48 h after* P.g. *LPS challenge (Figures 3(j) and 3(k)). These results suggest that an elevated level of CatB is positively associated with NF-*κ*B activation and oxidative damage after challenge with* P.g.* LPS.

Interestingly, the* P.g.* LPS-induced increase in levels of 8-OHdG and decrease in levels of I*κ*B*α* were significantly inhibited by CA-074Me at 48 h after* P.g.* LPS challenge (Figures 3(e)–3(i)). These observations demonstrate that CatB is involved in the proteolytic degradation of the I*κ*B*α* and oxidative DNA damage during chronic* P.g.* LPS challenge. Therefore, the novel mechanism of CatB in regulating the expression of collagens by fibroblasts is via chronically activating TLR2/NF-*κ*B signaling and subsequent oxidative damage.

### 3.4. Determination of CatB and Oxidative Damage in Fibroblasts of Inflamed Tissues with Chronic Periodontitis

**Figure 4:**
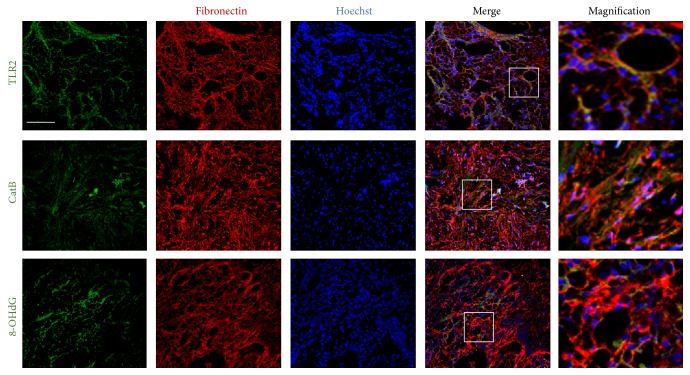
The immunofluorescent CLSM images of TLR2, CatB, and 8-OHdG (green) with fibronectin (red) and Hoechst-stained nuclei (blue) in the inflamed tissues of patients with chronic periodontitis. Scale bar = 50 *μ*m. The magnifical images are made from the square areas. The increased CatB is closely related to the oxidative damage during the chronic activation of TLR2/NF-*κ*B signaling.

**Figure 5:**
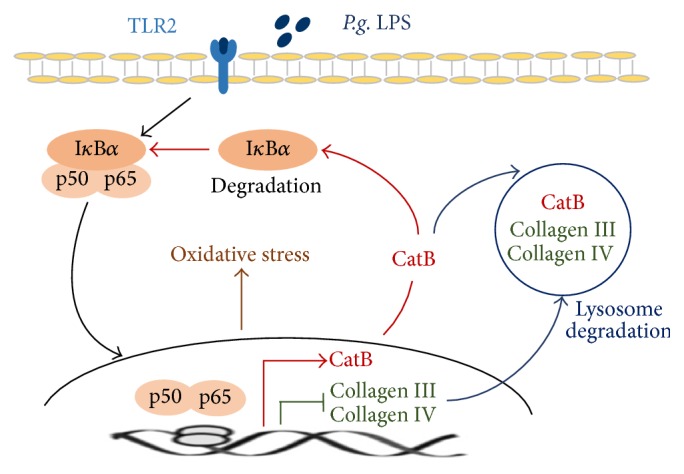
A schematic representation of the effects and the novel molecular mechanisms of CatB in regulating the expression of collagens III and IV via chronic activation of TLR2/NF-*κ*B signaling and subsequent oxidative damage.

TLR2, CatB, and 8-OHdG were expressed in the fibroblasts of inflamed tissues with chronic periodontitis and found to be localized in 89%, 87%, and 86% of fibronectin-positive fibroblasts, respectively (Figure 4). These results further demonstrate that the increased CatB is involved in oxidative damage in inflammatory tissues via the chronic activation of TLR2/NF-*κ*B signaling. The critical role and novel mechanism of CatB in regulating the expression of collagens by fibroblasts via this activation and oxidative damage were summarized in Figure 5.

## 4. Discussion

The major finding of the present study was clarifying the critical role of CatB in regulating collagen expression by fibroblasts via prolonging TLR2/NF-*κ*B activation (summarized in Figure 5). To our knowledge, this is the first study to demonstrate the involvement of CatB in collagen expression during chronic inflammation and oxidative stress, thereby adding to the available information regarding the mechanisms of delayed tissue repair during chronic inflammation.

We focused on collagens III and IV in fibroblasts because of their importance in wound healing and tissue remodeling. As the main fibrillar collagens in connective tissues [1], collagen III is suggested to regulate collagen I synthesis [2], since collagen III-deficit mice exhibit more pronounced wound contracture than those with collagen III [5], and upregulating collagen III expression using natural remedies accelerates cutaneous wound healing in rats [6]. However, the expression of collagen III is regulated by the activity of local fibroblasts [6]. Collagen IV, as a major component of the BM, participates in the inflammatory responses during tissue repair [7, 28, 29], principally via cellular regulation and migration [9, 30]. Indeed, Col4a1 (human collagen IV gene) mutation-associated phenotypic features include chronic inflammation and immune activation [10, 11].

In the present study, the significant increase in the mRNA expression of collagens III and IV in fibroblasts at 3 and 12 h (the early culture periods) might have been a definitive response to challenge with the pathogenic agent TLR2 agonist* P.g.* LPS during the acute activation of TLR2 signaling [27], further demonstrating the expression of TLR2 in fibroblasts [13, 14]. In contrast, the mRNA expressions of both collagens III and IV in fibroblasts were significantly decreased at 24 and 48 h (the late culture periods) after challenge with* P.g.* LPS, possibly due to the chronic activation of TLR2 signaling. The concentration of* P.g.* LPS (1 *μ*g/mL) used in the present experiments was one-thousandth of the concentration that reduced cell viability (1000 *μ*g/mL), indicating that the* P.g.* LPS-induced decreases in collagen expression occurred in living fibroblasts (Figure 1(a)).

Of note, the* P.g. *LPS-induced decreases in collagen III and IV reflected the* P.g.* LPS-induced decrease in expression of I*κ*B*α* and increase in expression of 8-OHdG. However, the* P.g.* LPS-induced decreases in expression of collagens were completely restored by Bay 11-7082 and SN50, the specific inhibitor of NF-*κ*B (Figures 3(a) and 3(b)), suggesting that these reductions in collagen expression were dependent on NF-*κ*B activation and oxidative damage, given that activated NF-*κ*B exacerbates oxidative stress [31]. These results are consistent with a previous finding that NF-*κ*B activation is necessary for UVC-decreased collagen biosynthesis [19] and that NF-*κ*B inhibits collagen I gene expression [18]. The present findings suggest that TLR2/NF-*κ*B-dependent decreases in the expression of collagen by fibroblasts may result in delayed tissue repair during chronic inflammation.

Elevated levels of CatB in fibroblasts are typically observed in many chronic inflammatory diseases, including rheumatoid arthritis as well as periodontitis [32–34]. Increased CatB expression in fibroblasts leads to tissue destruction, as fibroblasts are the prominent resident cells in the soft connective tissues [35]. In the present study, we determined that* P.g.* LPS increased the expression of CatB in fibroblasts, similar to the previous findings in macrophages [36]. Of note, the increased expression of CatB mRNA in the fibroblasts was observed at 24 and 48 h but not at 3 or 12 h after challenge with* P.g.* LPS, which agreed with the previous finding that CatB levels were not increased in the acute and intermediate stages of bacterial infection [37], implying that Cat B expression in fibroblasts might be induced by chronic activation of TLR2 signaling. Importantly, the* P.g.* LPS-induced increase in the CatB mRNA expression was paralleled by a* P.g.* LPS-induced decrease in the mRNA expression of both collagens III and IV, but this decrease was completely restored by pretreatment with CA-074Me. Surprisingly, the mean mRNA expression of collagens III and IV was significantly increased in the primary fibroblasts from CatB^−/−^ mice from 24 h after challenge with* P.g.* LPS. The findings demonstrate the critical role of CatB in regulating collagen expression by fibroblasts during chronic activation of TLR2 signaling. CatB in* P.g.* LPS-challenged fibroblasts may not be involved in apoptosis [38], as the cell viabilities were not reduced with a* P.g.* LPS-induced increase in the expression of CatB. In addition, we also detected the expression of CatB in fibroblasts in the inflamed periodontal tissues from patients with chronic periodontitis (Figure 4), which was consistent with the findings in patients with rheumatoid arthritis [39, 40], inflammatory bowel disease [41], and polymyositis [42]. However, neither collagen III nor collagen IV was detected in these inflamed periodontal tissues (data not shown), strongly suggesting that CatB critically regulates collagen expression by fibroblasts during chronic inflammation.

CatB was recently found to regulate NF-*κ*B activation [24]. However, conversely, CatB expression is believed to be NF-*κ*B-dependent as the promoter of CatB has NF-*κ*B binding site [43]. In almost all cell types, including fibroblasts, NF-*κ*B exists as a dimer of a p50 and p65 subunit that is retained in an inactive cytoplasmic complex by binding to the I*κ*B*α*. NF-*κ*B can be activated by TLR2 agonists as well as proinflammatory molecules, such as IL-1*β*, which induce phosphorylation acutely and proteolytic degradation of the I*κ*B subunit chronically [21]. In the present study, an analysis of the whole cell extracts showed that the* P.g.* LPS-induced I*κ*B*α* degradation in fibroblasts was paralleled by a* P.g. *LPS-induced increase in CatB in the late culture period; furthermore, the* P.g.* LPS-induced I*κ*B*α* degradation was prevented by CA-074Me pretreatment (Figure 3). These results coincide with our previous observation of CatB-dependent I*κ*B*α* degradation in microglia in a hypoxia/ischemia model [24] and strongly suggest that CatB is involved in the proteolytic degradation of the I*κ*B subunit for slowing the migration out of the NF-*κ*B complex from the nucleus, leading to sustained NF-*κ*B activation in response to chronic* P.g. *LPS challenge.

This is the first report to clarify the novel mechanism of CatB involved in the downregulation of collagens III and IV via chronic activation of TLR2/NF-*κ*B signaling. Furthermore, the* P.g.* LPS-induced increase in 8-OHdG might have been prevented by CA-074Me, suggesting the possible involvement of CatB in promoting oxidative stress through prolonging NF-*κ*B activation, as activated NF-*κ*B exacerbates oxidative stress [31].

CatB is considered a major lysosomal cysteine protease for the degradation of collagen in soft connective tissues, as it possesses both endopeptidase and exopeptidase activity which differs from other lysosomal cysteine proteases [44]. The increased expression of CatB by the chronic activation of TLR2/NF-*κ*B signaling may also be involved in degrading the intercellular and extracellular collagen produced by fibroblasts, as CatB activities are required for the degradation of intracellular and extracellular collagen IV [45]. Furthermore, CatB is known to activate other collagenolytic matrix metalloproteinases for collagen degradation in fibroblasts at elevated levels during chronic inflammation [46].

## 5. Conclusion

CatB regulates the expression of collagens III and IV by fibroblasts via prolonging TLR2/NF-*κ*B activation and oxidative stress (schematic illustration in Figure 5). Considering the role of CatB in collagen expression, CatB-specific inhibitors may be a useful approach for improving inflammation-delayed connective tissue repair, such as that found in dermatitis and periodontitis.
